# Comparisons of Metastatic Patterns, Survival Outcomes and Tumor Immune Microenvironment Between Young and Non-Young Breast Cancer Patients

**DOI:** 10.3389/fcell.2022.923371

**Published:** 2022-07-14

**Authors:** Hengwen Sun, Wei Huang, Fei Ji, Yi Pan, Lu Yang

**Affiliations:** ^1^ Department of Radiation Oncology, Guangdong Provincial People’s Hospital, Guangdong Academy of Medical Sciences, Guangzhou, China; ^2^ Cancer Center, Department of Breast Cancer, Guangdong Provincial People’s Hospital, Guangdong Academy of Medical Sciences, Guangzhou, China

**Keywords:** age-related breast cancer, metastatic pattern, survival prognosis, tumor immune microenvironment, immunotherapy

## Abstract

**Background:** Metastases are the main cause of breast cancer-related deaths. Breast cancer has a more aggressive phenotype and less favorable prognosis in young females than in older females. In this study, we aimed to compare the metastatic patterns, survival outcomes and tumor immune microenvironment of young and non-young breast cancer patients.

**Methods:** Patients with a diagnosis of breast cancer were identified from the Surveillance, Epidemiology, and End Results (SEER) database between 2010 and 2015. The significance of young age (≤40 years) in the metastatic profile and prognosis of breast cancer was investigated. The transciptome expression data were acquired from The Cancer Genome Atlas (TCGA) database. And the differentially expressed genes (DEGs) and primarily enriched function pathways were identified by comparing between young and non-young breast cancer samples, and tumor immune infiltrating cell types in the tumor microenvironment were compared.

**Results:** A total of 281,829 female breast cancer patients were included in SEER: 18,331 young (6.5%) and 263,498 non-young (93.5%) women. The metastatic rates of bone, liver and distant lymph nodes (DLNs) in the young cohort were significantly higher than those in the non-young cohort. The most frequent two-site metastatic combination was bone and liver (0.61%) in the young cohort, whereas it was bone and lung (0.32%) in the non-young cohort. Breast cancer-specific survival (BCSS) was significantly shortened among those in the young cohort compared with those in the non-young cohort (*p* < 0.001). Young age was associated with significantly shorter BCSS only among patients with HR+/HER2- tumors (*p* < 0.001). The enriched biological pathways based on DEGs between two cohorts were related to the regulation of immune response and several metabolic processes. M2 macrophages were significantly abundant in non-young breast cancer than young breast cancer.

**Conclusion:** Young and non-young breast cancer patients present with different metastatic patterns. Young age is a negative prognostic factor, particularly for HR+/HER2- breast cancer. The differences in metastatic patterns between young and non-young cohorts should be taken into account in the clinical management of metastatic breast cancer. The young breast cancer patients may gain better response to immunotherapy due to immune activated TME than non-young breast cancer.

## Introduction

Breast cancer has surpassed lung cancer as the most commonly diagnosed cancer worldwide, accounting for 11.7% of all cancer cases (Sung et al., 2021). For women, breast cancer accounts for 1 in 4 of all cancer cases and for 1 in 6 of cancer-related deaths. It is estimated that approximately 90% of breast cancer-related deaths are attributed to metastasis, suggesting that metastases are the main causes of breast cancer-related deaths (Cancer Genome Atlas Network, 2012). Despite substantial advances in treatment in recent years, 20%–30% of patients with early-stage breast cancer will experience recurrence with distant metastatic disease, and the prognosis for metastatic breast cancer remains poor (Early Breast Cancer Trialists' Collaborative Group, 2005; Mego et al., 2010). Lack of insight into the mechanisms behind metastasis poses challenges to the development of antitumor therapeutics.

Metastasis-initiating cells can struggle to survive in an unfamiliar microenvironment that is distinctively changed from the primary tumor and expand their daughter cells at secondary locations (Yang et al., 2020). Breast cancer is prone to metastasis to several distinct organs, including bone, lung, liver, and brain (Liang et al., 2020). Metastatic heterogeneity substantially refrains from eradicating metastatic diseases. The metastatic incidence of breast cancer varies with the molecular subtype of this disease. Breast cancer patients have a bone metastasis incidence of > 30%, making bone the most frequent site of metastases in all subtypes except triple-negative tumors (Kennecke et al., 2010). While human epidermal growth factor receptor-2 (HER2)-positive tumors have a significantly higher metastatic rate to the brain, liver and lung, patients with triple-negative tumors have a higher rate of brain, lung and distant nodal metastases (Foulkes et al., 2010; Kennecke et al., 2010). Therefore, the metastatic behavior of different breast cancer subtypes may differ from each other.

Young breast cancer is typically defined as occurring in patients aged ≤ 40 years at the diagnosis of breast cancer. Annually, approximately 11,000 women aged under 40 are diagnosed with breast cancer in the United States, and breast cancer is the leading cause of cancer deaths for young women (Freedman and Partridge, 2017). There was a small but statistically significant increase (2% per year) in the incidence of breast cancer in the United States for women aged 25–39 years (Johnson et al., 2013). The GRELL study in 7 countries from Europe showed that the incidence of breast cancer in women under 40 increased 1.2% annually, especially for women under 35 years of age (Leclère et al., 2013). Compared with Western white women, breast cancer occurs at a young median age (mean: 45–55 years) in Chinese women (Fan et al., 2014). Typically, breast cancer in young patients is characterized by a higher risk of recurrence, less favorable prognosis, poorer treatment response, and more aggressive phenotypes (Colleoni et al., 2002; Anders et al., 2008; Narod, 2012). Young breast cancer patients are more likely to have higher-grade, triple-negative and HER2-positive tumors than older women (Azim et al., 2012; Azim and Partridge, 2014; Rosenberg et al., 2015). Thus, young breast cancer has become a heated issue in the field of cancer research.

It is well established that aging is associated with declining immune function, which is known as immunosenescence (Pawelec and Solana, 1997) and accompanied by the beginning of a state of low-grade chronic inflammation named “inflammaging” (Franceschi et al., 2000). Besides, this dysregulated response may impact on the pathogenesis of severe age-related diseases including cancer. The tumor microenvironment, a complex collection of cells including fibroblasts, epithelial cells, adipose cells, immune cells (i.e., neutrophils, monocytes/macrophages, dendritic cells, Treg cells and other lymphocytes) and the extracellular matrix (Jackaman et al., 2009), plays a pivotal role in tumor immune evasion and it may be altered during aging as a result of age-related immune dysfunction. For example, Treg cells are reported to increase in lymphoid tissues during aging and likely suppress the development of anti-tumor T cell responses through secretion of TGF-β and IL-10 (Franceschi et al., 2017). Additionally, mammary tumors in elderly mice have lower numbers of infiltrating CD4+ and CD8+ T cells compared to younger mice (Provinciali et al., 2000).

However, the metastatic patterns, survival of breast cancer and the abundances of immune infiltrating cells in TME between young and non-young women have not been comprehensively described. The objectives of this study were to determine the differences in metastatic patterns, survival outcomes and immune infiltrating cells in TME between young and non-young breast cancer patients based on the Surveillance, Epidemiology, and End Results (SEER) program and The Cancer Genome Atlas (TCGA) database.

## Materials and Methods

### Study Population

**FIGURE 1:**
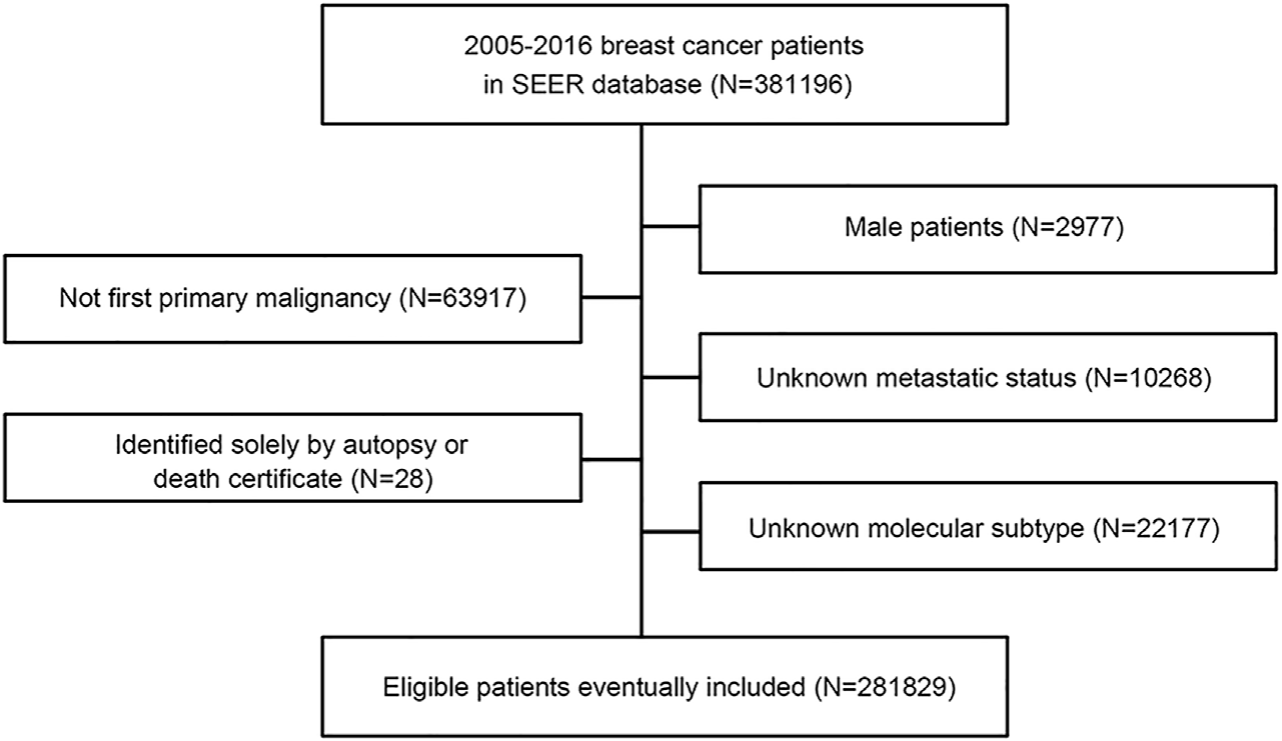
Flowchart of the inclusion of patients in SEER database.

This study used the SEER database, which collects information on patients with cancer from SEER cancer registries in the United States (US), covering approximately 28% of the US population (Cronin et al., 2014). From 1 January 2010, to 31 December 2015, patients with a diagnosis of breast cancer were identified from the SEER database with SEER∗Stat version 8.3.9 (https://seer.cancer.gov/). We collected patients from 2010 to 2015 because the recording of human epidermal growth factor receptor-2 (HER2) status began in 2010, and patients had relatively long-term follow-up. The variables that we extracted from the SEER database included age, sex, race, laterality, histologic type ICD-O-3, grade, T stage, N stage, M stage, TNM stage (AJCC stage group 6th edition), estrogen receptor (ER) status, progesterone receptor (PR) status, HER2 recode (2010+), breast subtype (2010+), CS mets at dx (2004–2015), first malignant primary indicator, survival months flag, vital status, COD to site rec KM and survival months. The flowchart of the SEER data screening is illustrated in Figure 1. Young breast cancer is defined as occurring in patients aged ≤ 40 years at the diagnosis of breast cancer.

Patients were included according to the following criteria: 1) female patients; 2) patients with first primary breast cancer; 3) record of metastatic status; and 4) record of estrogen receptor (ER), progesterone receptor (PR), and HER2 status. Patients who did not meet the inclusion criteria were excluded. We also excluded patients who were identified solely by autopsy or death certificate. Because the SEER database is available to the public, use of the data does not require ethical approval.

Additionally, the transciptome RNA-seq expression data together with detailed clinicopathological information were obtained from The Cancer Genome Atlas (TCGA) database, including 100 young breast cancer samples and 997 non-young breast cancer samples.

### Identification of Differentially Expressed Genes and Implementation of Function Enrichment Analysis Between Young and Non-Young Breast Cancer

To identify the DEGs between young and non-young breast cancer samples, “edgR” R package (McCarthy et al., 2012) was utilized in TCGA-BRCA cohort with the significance criteria set to |log2FC| > 1 and False Discovery Rate (FDR) < 0.05. A volcano plot and a heatmap of these remarkable DEGs were portrayed. To further uncover potential regulatory interactions and hub genes among these DEGs, a protein-protein interaction (PPI) network was forged *via* the STRING database (Szklarczyk et al., 2019) and depicted in Cytoscape (version 3.9.1) (Shannon et al., 2003).

To investigate the pathways enriched in the age-related breast cancer groups, we performed Kyoto Encyclopedia of Genes and Genomes (KEGG) pathway analysis and Gene Ontology (GO) analysis by applying a threshold *p*-value < 0.05, minimum count of 5, and enrichment factor > 0.15 through implementing the clusterProfiler R package (Yu et al., 2012).

### Comparison of Immune Cell Infiltration Between Two Breast Cancer Groups

The CIBERSORT deconvolution algorithm (Newman et al., 2015) was utilized to calculate the abundance of 22 tumor immune infiltrating cell types and xCell algorithm (Aran et al., 2017) was also performed to formulate the distribution of 64 immune cell types and stromal cell types in the tumor microenvironment based on the gene expression matrix of TGCA-BRCA cohort of young and non-young breast cancer samples. A violin plot was displayed to unveil the results of CIBERSORT and xCell analysis while *p* < 0.05 from Wilcox test was considered statistically significant. Besides, correlation scatter diagrams were depicted to show the correlation between age and several tumor immune cells based on the result of CIBERSORT analysis through Spearman’s correlation test.

### Statistical Analysis

Patient characteristics and metastatic patterns were analyzed descriptively, and differences in categorical variables between different groups were compared with the chi-square test and Fisher’s exact test. Kaplan–Meier curves were drawn for overall survival (OS) and breast cancer-specific survival (BCSS), and differences were compared by the log-rank test. Univariate analysis was performed with variables including being young or not, race, histology, grade, T stage, N stage, and metastasis status of bone, brain, liver, lung, and distant lymph node (DLN). Variables reaching a significance level of 0.05 were included in the multivariable analysis to determine the independent prognostic factors. Hazard ratios and their two-sided 95% confidence intervals (CIs) were estimated by Cox proportional hazards regression analysis. All statistical tests were two-sided, and *p* values less than 0.05 were considered statistically significant. Analyses were conducted using R (version 4.0.4, Vienna, Austria) and GraphPad Prism (version 8.0.1.244, San Diego, CA, United States).

## Results

### Patient Characteristics in Surveillance, Epidemiology, and End Results

A total of 281,829 female patients were diagnosed with breast cancer between 2010 and 2015 in the SEER database and included in this study. This cohort was composed of 18,331 young (6.5%) and 263,498 non-young (93.5%) women. Table 1 illustrates the patient characteristics, which were divided into young and non-young cohorts. Tumor characteristics differed by age. Compared with the non-young cohort, the young cohort had a high incidence of being black, Asian or Pacific Islander race, having right breast disease, invasive carcinoma histology, poor grade, high TNM stage, hormone receptor (HR) +/HER2 + and HER2-enriched and triple negative subtypes.

**TABLE 1:** Patient characteristics of the young cohort and the non-young cohort.

	Young cohort (*n* = 18,331)	Non-young cohort (*n* = 263,498)	*p* value
Race			<0.001
White	12951 (70.7%)	208730 (79.2%)	
Black	2761 (15.1%)	28926 (11.0%)	
Asian or Pacific Islander	2319 (12.7%)	22873 (8.7%)	
American Indian/Alaska Native	143 (0.8%)	1576 (0.6%)	
Unknown	157 (0.9%)	1393 (0.5%)	
Lateral			0.007
Left	9153 (49.9%)	133447 (50.6%)	
Right	9162 (50.0%)	129583 (49.2%)	
Both	3	66	
Unknown	13 (0.1%)	402 (0.2%)	
Histology (ICD-O-3)			<0.001
Invasive carcinoma	16770 (91.5%)	222165 (84.3%)	
Favorable	314 (1.7%)	7743 (2.9%)	
Metaplastic	93 (0.5%)	1147 (0.4%)	
Others	1154 (6.3%)	32443 (12.3%)	
Grade			<0.001
I	1518 (8.3%)	60239 (22.9%)	
II	6221 (33.9%)	112680 (42.8%)	
III	9647 (52.6%)	78555 (29.8%)	
IV	82 (0.4%)	700 (0.3%)	
Unknown	863 (4.7%)	11324 (4.3%)	
Stage, AJCC 6th			<0.001
0	5	72	
I	4770 (26.0%)	127432 (48.4%)	
II	8694 (47.4%)	91978 (34.9%)	
III	3526 (19.2%)	29353 (11.1%)	
IV	1031 (5.6%)	11009 (4.2%)	
Unknown	305 (1.7%)	3654 (1.4%)	
T stage			<0.001
Tis	5	72	
1	7028 (38.3%)	154511 (58.6%)	
2	7815 (42.6%)	77740 (29.5%)	
3	2129 (11.6%)	15686 (6.0%)	
4	925 (5.0%)	10299 (3.9%)	
Unknown	429 (2.3%)	5190 (2.0)	
N stage			<0.001
0	8902 (48.6%)	176507 (67.0%)	
1	6384 (34.8%)	58600 (22.2%)	
2	1525 (8.3%)	13224 (5.0%)	
3	1167 (6.4%)	10641 (4.0%)	
Unknown	353 (1.9%)	4526 (1.7%)	
M stage			<0.001
0	17210 (93.9%)	251697 (95.5%)	
1	1121 (6.1%)	11793 (4.5%)	
Unknown	0	8	
Molecular Subtypes			<0.001
HR+/HER2−	10168 (55.5%)	195427 (74.2%)	
HR+/HER2+	3441 (18.8%)	27339 (10.4%)	
HER2-enriched	1307 (7.1%)	12108 (4.6%)	
Triple negative	3415 (18.6)	28624 (10.9%)	

Abbreviation: AJCC, American joint committee on ancer; HR, hormone receptor; HER2, human epidermal growth factor receptor-2.

By the time of diagnosis, 12,355 patients (4.4%) were recorded as having at least distant metastasis in all patients. The top 5 most commonly diagnosed metastatic sites were bone (8,623, 3.1%), lung (3991, 1.4%), liver (3,213, 1.1%), distant lymph node (3,203, 1.1%), and brain (869, 0.3%), which accounted for 95.7% (12,355/12,914) of all metastatic cases.

### Metastatic Patterns of Young and Non-Young Cohorts

**FIGURE 2:**
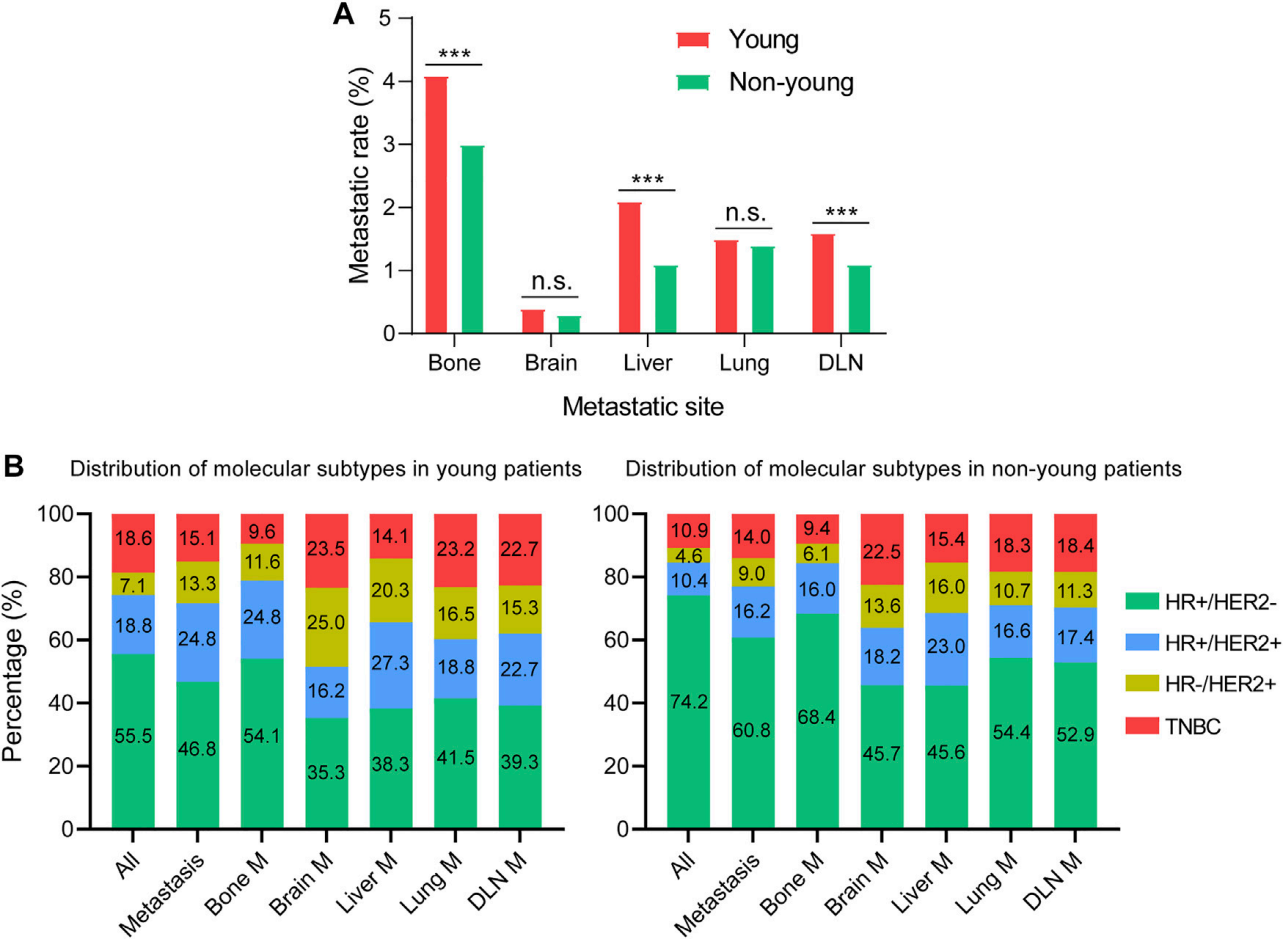
Metastatic patterns of young and non-young cohorts. **(A)** Comparison of the rates of different metastatic sites. **(B)** Distribution of molecular subtypes in different metastatic cohorts. ***p < 0.001; n.s., no significance. HR, hormone receptor; HER2, human epidermal growth factor receptor 2; TNBC, triple negative breast cancer; M, metastasis; DLN, distant lymph node.

**FIGURE 3:**
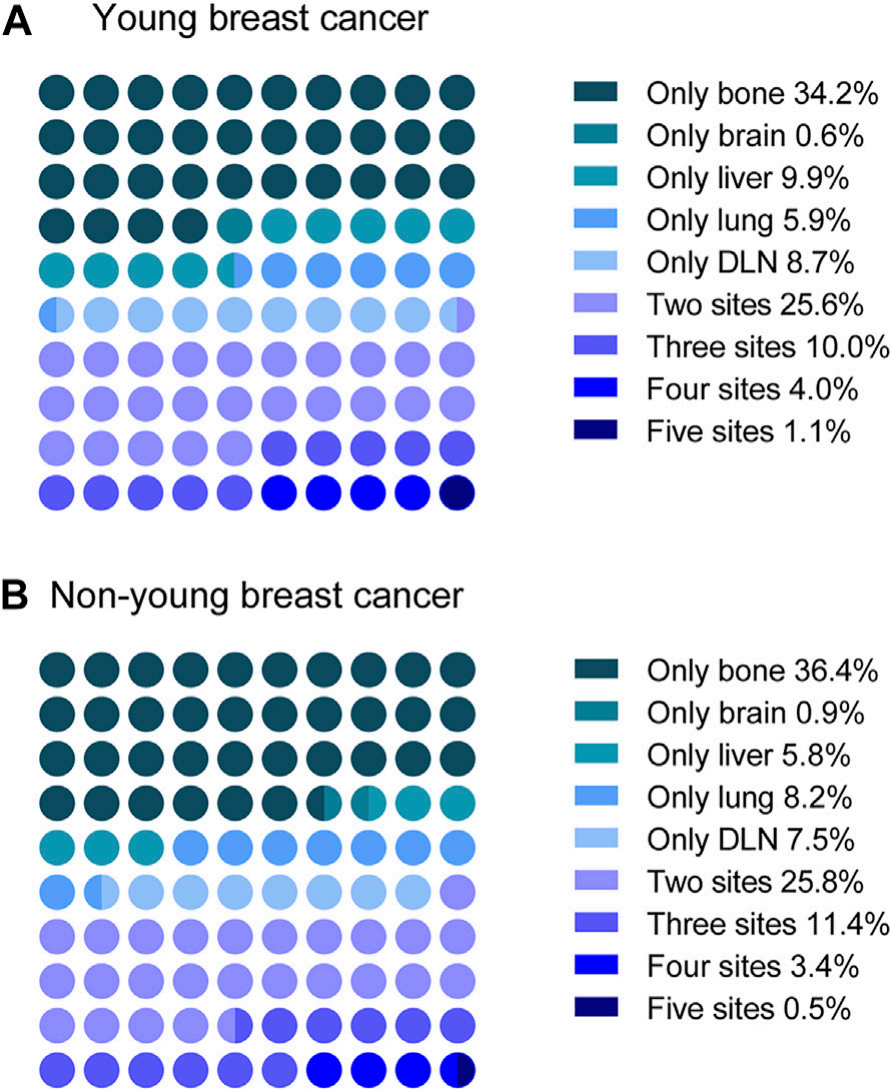
Distribution of single-site metastasis and multisite metastases in **(A)** young and **(B)** non-young patients. DLN, distant lymph node.

The rates of different metastatic sites were compared between young and non-young cohorts. The metastatic rates of bone, liver and DLN in the young cohort were significantly higher than those in the non-young cohort, whereas there were no significant differences in the metastatic rates of brain and lung between young and non-young cohorts (Figure 2A). We further investigated the impact of molecular subtypes on the metastatic sites in young and non-young patients (Figure 2B). For all patients with metastasis, the percentage of the HR+/HER2- subtype was much lower in the young cohort (46.8%) than in non-young cohort (60.8%). For the young cohort, the percentage of HR+/HER2- was highest among patients with bone metastasis (54.1%) and gradually decreased in patients with lung (41.5%), DLN (39.3%), liver (38.3%) and brain (35.3%) metastases. The same trend for the HR+/HER2– subtype was found in the non-young cohort. The percentages of HER2+ (HR+/HER2+ and HER2-enriched) subtypes were high among patients with liver and brain metastasis in both young and non-young cohorts (Figures 3A,B). In addition, the percentage of triple-negative breast cancer (TNBC) was significantly increased in patients with brain (23.5%), lung (23.2%), DLN (22.7%), and liver (14.1%) metastasis compared with those with bone (9.6%) metastasis in the young cohort. In a similar manner, the percentage of TNBC was significantly higher in patients with brain (22.5%), lung (18.3%), DLN (18.4%), and liver (15.4%) metastasis than in those with bone (9.4%) metastasis in the non-young cohort.

### Multisite Metastasis and Co-Metastasis

Since patients may have more than one metastatic site at diagnosis, we next explored the distribution of multisite metastasis and co-metastasis. As shown in Figure 3, multisite metastasis accounted for 40.7% and 41.1% of all cases with metastasis in the young and non-young cohorts, respectively. For multisite metastasis, two-site metastasis (25.6%) was the most common pattern for young patients, followed by three-site (10.0%), four-site (4.0%), and five-site (1.1%) metastasis. In a similar manner, the percentage of multisite metastasis was decreased along with the increase in metastatic site number for non-young patients, with two-site metastasis (25.8%) being the most common pattern, followed by three-site (11.4%), four-site (3.4%), and five-site (0.5%) metastasis.

We further compared the specific metastatic patterns of the five metastatic sites between young and non-young cohorts (Table 2). The most frequent two-site metastatic combination was bone and liver (0.61%) for the young cohort, whereas it was bone and lung (0.32%) for the non-young cohort. For three-site metastasis, the most common combination was bone, liver and DLN (0.18%) for the young cohort, while the most common combination was bone, liver and lung (0.14%) for the non-young cohort. The most frequent four-site combination was bone, liver, lung and DLN in both the young (0.16%) and non-young (0.09%) cohorts.

**TABLE 2:** Frequencies of metastatic pattern.

Metastatic pattern	Young cohort			Non-young cohort			*p* value
	Number	(%) in whole cohort	(%) in M1 cohort	Number	(%) in whole cohort	(%) in M1 cohort	
One site							<0.001
Only bone	373	2.035	33.274	4104	1.558	34.800	
Only brain	7	0.038	0.624	106	0.040	0.899	
Only liver	108	0.589	9.634	650	0.247	5.512	
Only lung	64	0.349	5.709	921	0.350	7.810	
Only DLN	95	0.518	8.475	849	0.322	7.199	
Two sites							<0.001
Bone and brain	10	0.055	0.892	159	0.060	1.348	
Bone and liver	111	0.606	9.902	713	0.271	6.046	
Bone and lung	52	0.284	4.639	833	0.316	7.064	
Bone and DLN	53	0.289	4.728	498	0.189	4.223	
Brain and liver	2	0.011	0.178	17	0.006	0.144	
Brain and lung	4	0.022	0.357	51	0.019	0.432	
Brain and DLN	3	0.016	0.268	23	0.009	0.195	
Liver and lung	14	0.076	1.249	199	0.076	1.687	
Liver and DLN	11	0.060	0.981	94	0.036	0.797	
Lung and DLN	20	0.109	1.784	318	0.121	2.697	
Three sites							0.001
Bone and brain and liver	10	0.055	0.892	50	0.019	0.424	
Bone and brain and lung	0	0.000	0.000	82	0.031	0.695	
Bone and brain and DLN	3	0.016	0.268	40	0.015	0.339	
Bone and liver and lung	29	0.158	2.587	379	0.144	3.214	
Bone and liver and DLN	32	0.175	2.855	209	0.079	1.772	
Bone and lung and DLN	22	0.120	1.963	373	0.142	3.163	
Brain and liver and lung	2	0.011	0.178	19	0.007	0.161	
Brain and liver and DLN	1	0.005	0.089	9	0.003	0.076	
Brain and lung and DLN	0	0.000	0.000	27	0.010	0.229	
Liver and lung and DLN	10	0.055	0.892	96	0.036	0.814	
Four sites							0.196
Bone and brain and liver and lung	11	0.060	0.981	72	0.027	0.611	
Bone and brain and liver and DLN	1	0.005	0.089	23	0.009	0.195	
Bone and brain and lung and DLN	2	0.011	0.178	50	0.019	0.424	
Bone and liver and lung and DLN	30	0.164	2.676	226	0.086	1.916	
Brain and liver and lung and DLN	0	0.000	0.000	12	0.005	0.102	
Fiver sites							
Bone and brain and liver and lung and DLN	12	0.065	1.070	61	0.023	0.517	

DLN, distant lymph node.

**FIGURE 4:**
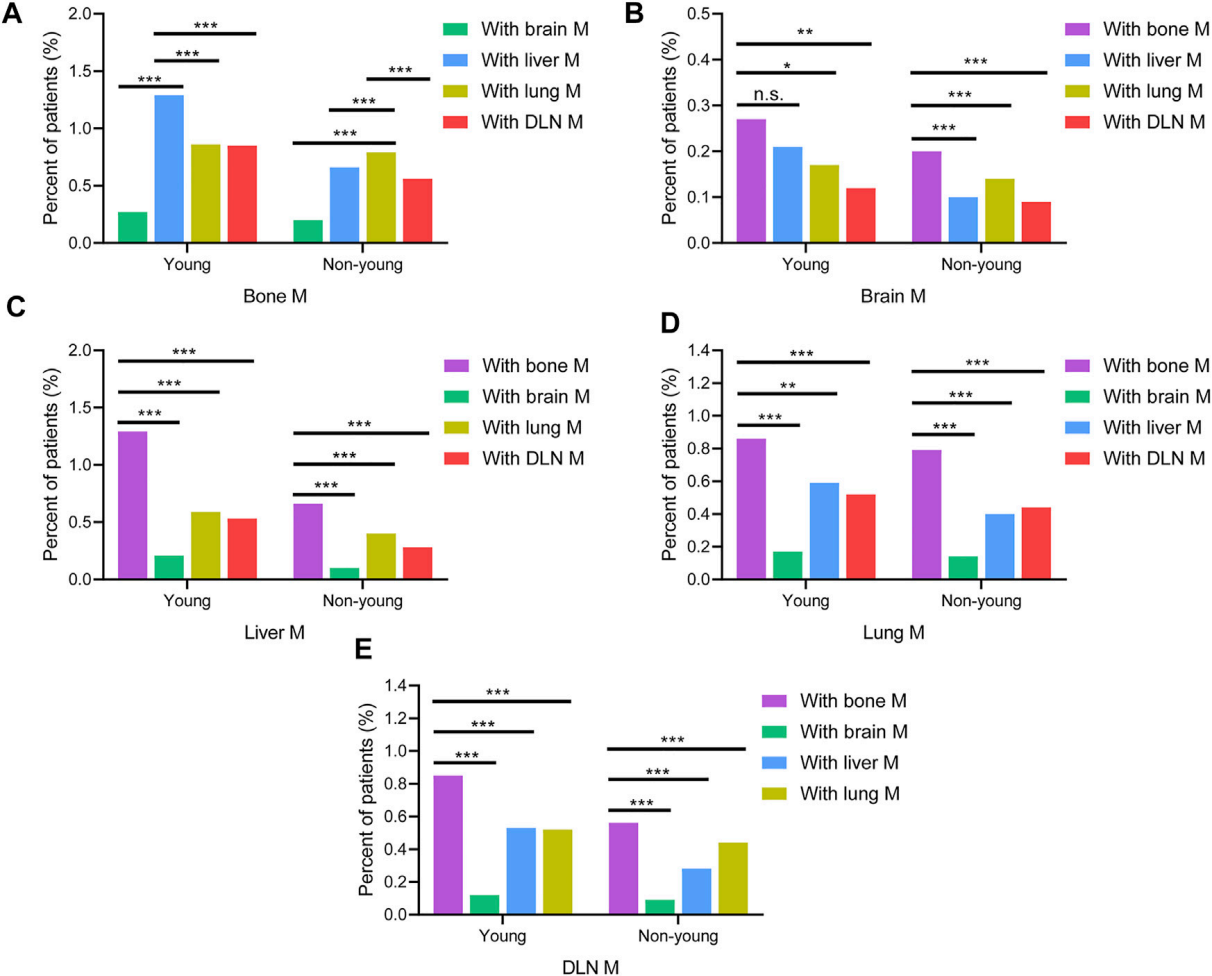
Comparison of the rates of co-metastatic sites with **(A)** bone M, **(B)** brain M, **(C)** liver M, **(D)** lung M, **(E)** DLN M between young and non-young cohorts. M, metastasis; DLN, distant lymph node.

Moreover, the pairwise interaction of the five metastatic sites was analyzed (Figures 4A–E). Young patients with bone metastasis had a higher co-metastasis rate of the liver (1.29%) than the lung (0.86%), DLN (0.85%), and brain (0.27%). However, for non-young patients with bone metastasis, the co-metastasis rate of the lung (0.79%) was significantly higher than that of the liver (0.66%), DLN (0.56%), and brain (0.20%). For patients with brain, liver, lung and DLN metastasis, the co-metastasis rate of bone was much higher than that of other sites.

Additionally, we performed the analysis of metastatic patterns in each T stage cohort separately. The results showed that the rate of multiple metastases was gradually increased from T1 to T4 in both young and non-young cohorts (Supplementary Figures S1A,B). The metastatic rates of bone, brain, liver, lung, and DLN were gradually increased from T1 to T4 in patients with single-site and multi-site metastases in both young and non-young cohort (Supplementary Figures S1C–F).

### Survival Outcomes

**FIGURE 5:**
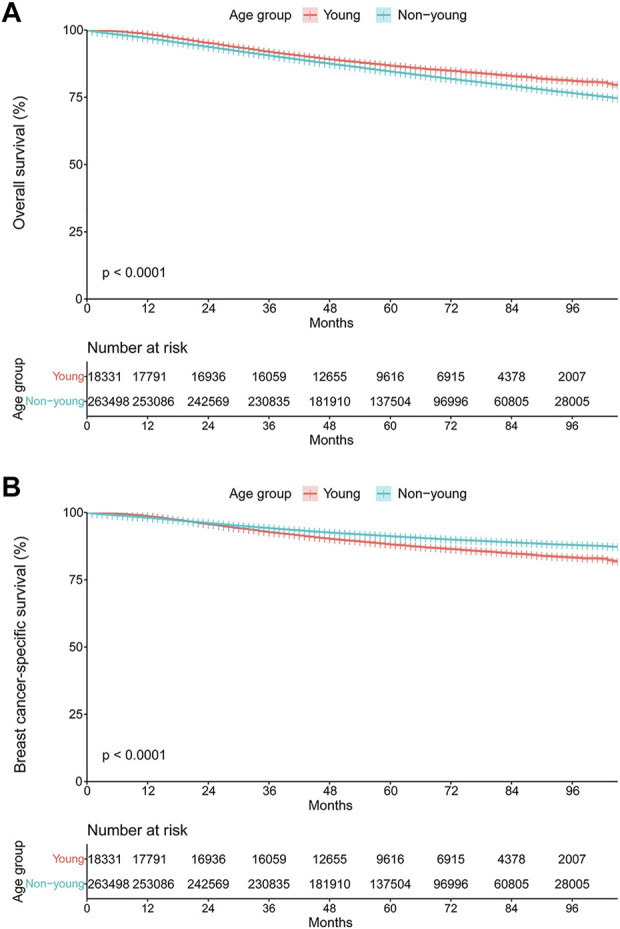
Kaplan–Meier curves of **(A)** overall survival and **(B)** breast cancer-specific survival stratified by age group.

**FIGURE 6:**
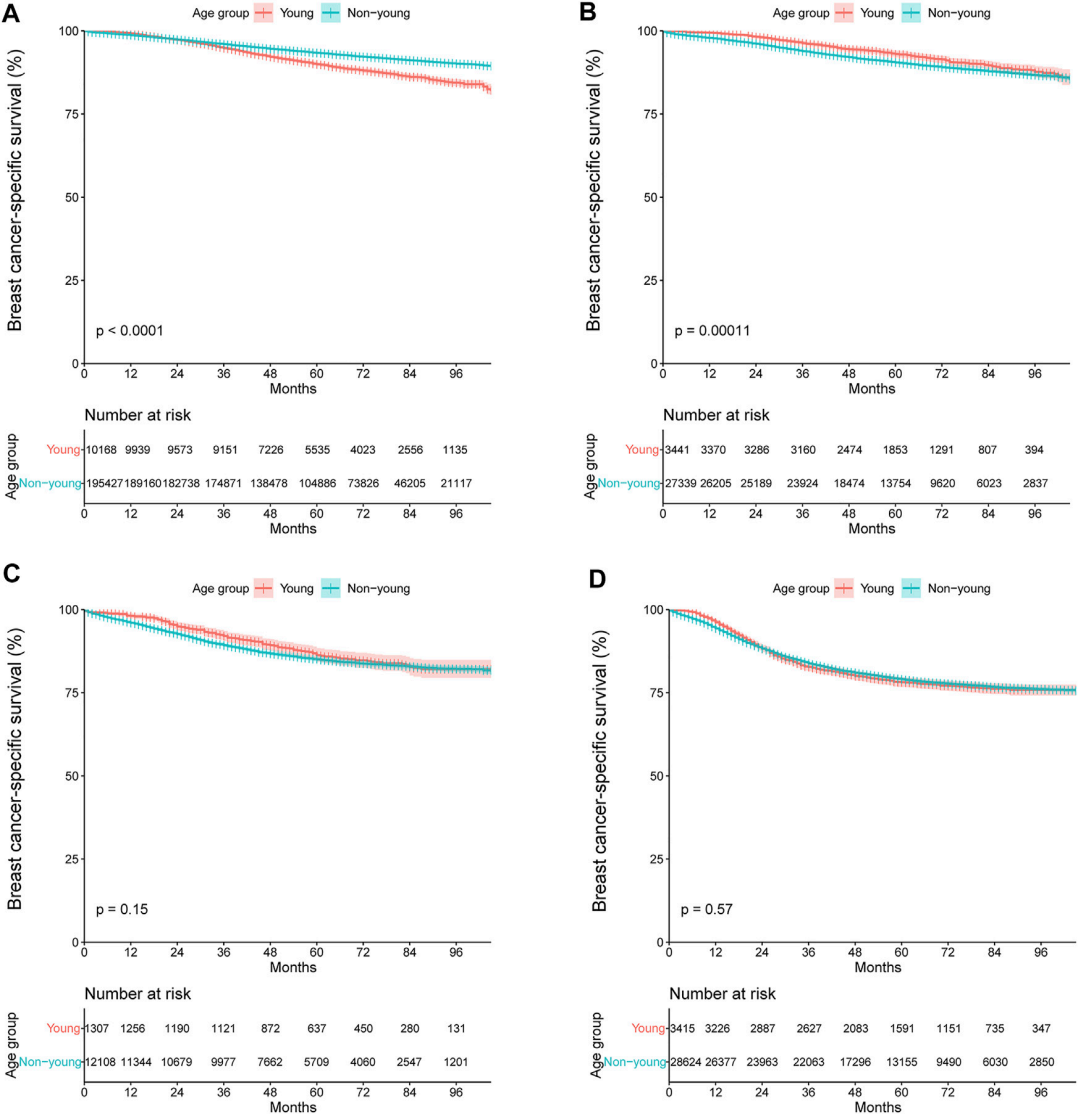
Kaplan–Meier curves of breast cancer-specific survival stratified by age group for **(A)** HR+/HER2-, **(B)** HR+/HER2+, **(C)** HER2-enriched, and **(D)** triple-negative breast cancer. +, positive; −, negative; HR, hormone receptor; HER2, human epidermal growth factor receptor 2.

The median follow-up was 68.1 (95% CI, 68.0–68.2) months for the entire study population. The OS and BCSS curves stratified by age group are shown in Figures 6A,B. Surprisingly, the young cohort had significantly lengthened OS compared with the non-young cohort (hazard ratio, 0.81; 95% CI, 0.78–0.85; *p* < 0.001; Figure 5A). However, BCSS was significantly shortened among those in the young cohort compared with the non-young cohort (hazard ratio, 1.36; 95% CI, 1.31–1.42; *p* < 0.001; Figure 5B). The 3-year and 5-year BCSS rates were 92.7% and 88.1% for patients in the young cohort and 94.1% and 91.2% for patients in the non-young cohort, respectively.

We further compared the BCSS between the young and non-young cohorts in different molecular subtypes (Figures 6A–D). The results showed that young age was associated with significantly shorter BCSS among patients with HR+/HER2- tumors (hazard ratio, 1.55; 95% CI, 1.45–1.64; *p* < 0.001; Figure 6A), but not among those with HER2-enriched (hazard ratio, 0.89; 95% CI, 0.76–1.05; *p* = 0.15; Figure 6C) or triple-negative tumors (hazard ratio, 1.02; 95% CI, 0.95–1.11; *p* = 0.57; Figure 6D). The 3-year and 5-year BCSS rates were 94.8% and 89.9% among the HR+/HER2- patients in the young cohort and 96.0% and 93.4% among those in the non-young cohort, respectively. Notably, for patients with HR+/HER2+ tumors, young patients had significantly longer BCSS than those who were not young (hazard ratio, 0.78; 95% CI, 0.69–0.89; *p* < 0.001; Figure 6B).

The association of BCSS with clinicopathologic characteristics for patients with HR+/HER2- tumors was further analyzed with univariate and multivariate analyses (Supplementary Table S1). In the univariate analysis, young/non-young, race, histology, grade, T stage, N stage, and metastatic status of bone, brain, liver, lung, and DLN were identified as significant prognostic factors. When these variables were further analyzed in the multivariate analysis, we found that young/non-young, grade, T stage, N stage, and metastatic status of bone, brain, liver, and lung had significant hazard ratios, indicating that they were significant predictors of BCSS.

### Investigation of Differentially Expressed Genes and Function Enrichment Analysis Between Young and Non-Young Breast Cancer

**FIGURE 7:**
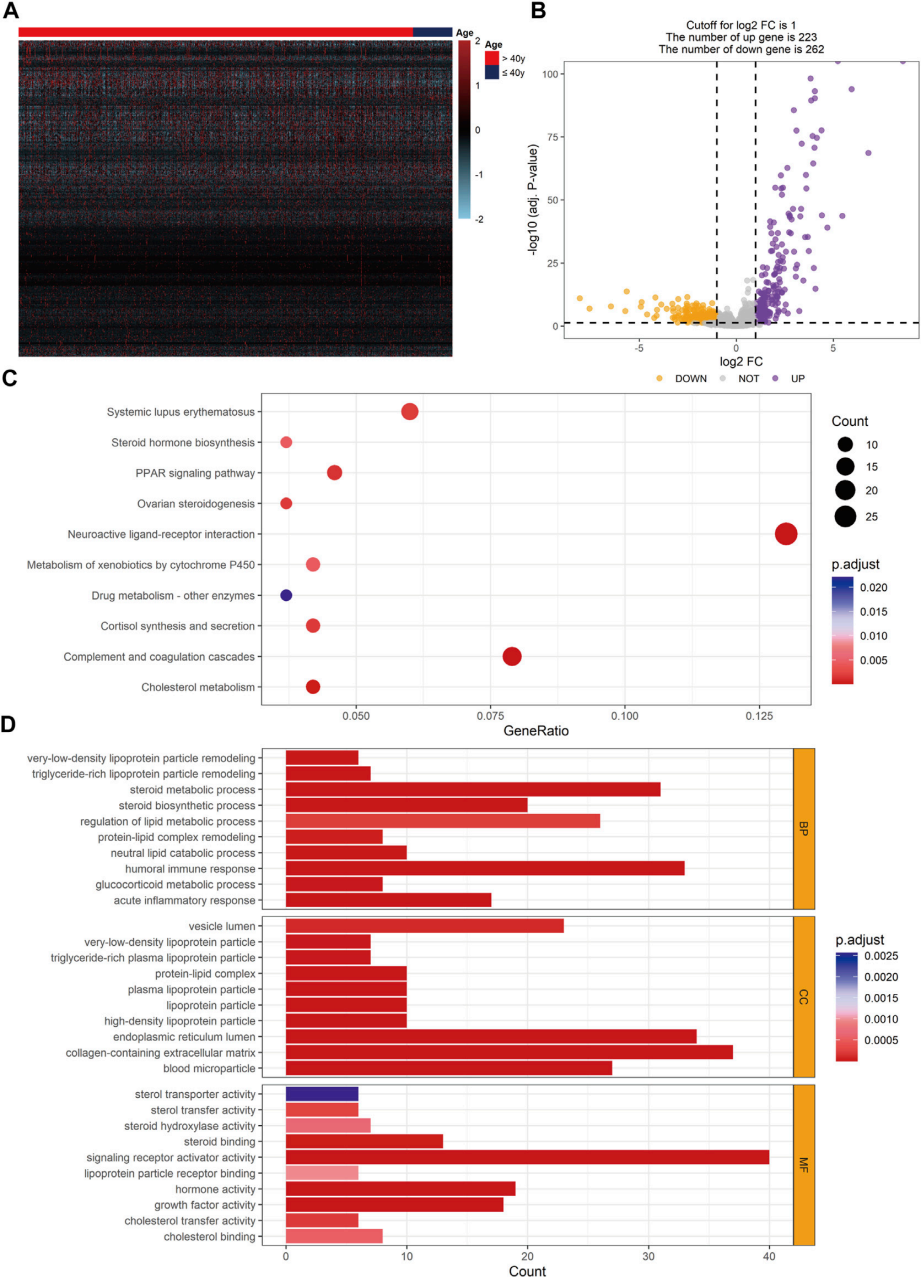
Identification of DEGs and enriched function pathways between young and non-young cohorts. **(A)** Heatmap of DEGs in TCGA-BRCA database. **(B)** Volcano plot exhibiting 485 DEGs. **(C)** KEGG analysis result based on DEGs. **(D)** GO analysis result based on DEGs. DEG, differentially expressed gene.

To discover the DEGs between young and non-young breast cancer samples, we compared the RNA-seq data of 100 young breast cancer samples and 997 non-young breast cancer samples in TCGA-BRCA database and found 485 DEGs using the threshold of |log2FC| > 1 and FDR < 0.05, including 223 significantly promoted genes and 262 essentially down-regulated genes in non-young breast cancer patients (Figures 7A,B). To further analyze the hub genes and potential regulatory interactions among these DEGs, a PPI network using the top 10 hub genes was formulated in Supplementary Figure S2A. The hub genes encompassed apolipoproteins (like APOA1, APOB, APOC3 and APOH), coagulation factors (such as FGA, FGB, FGG and F2), alpha-1-microglobulin and bukinin precursor (AMBP), alpha-2-HS-glycoprotein (AHSG), which indicated theses hub genes may have significant effect to the pathogenesis of age-related breast cancer. Subsequently, to further explore the potential biological differences and pathways occurred in age-related breast cancer, KEGG analysis by employing DEGs showed that the DEGs were enriched in complement and coagulation cascades, neuroactive ligand-receptor interaction, cholesterol metabolism, PPAR signaling pathway, cortisol synthesis and secretion, systemic lupus erythematosus and so on (Figure 7C). Moreover, GO analysis of biological processes showed that the DEGs were enriched in steroid metabolic process, humoral immune response, acute inflammatory response and regulation of lipid metabolic process. Cellular component analysis indicated that DEGs were abundant in collagen-containing extracellular matrix, high-density lipoprotein particle, plasma lipoprotein particle and lipoprotein particle. Molecular function analysis revealed that DEGs were primarily located in signaling receptor activator activity, hormone activity, growth factor activity, steroid binding and cholesterol transfer activity (Figure 7D). These signaling pathways are mainly relevant to regulation of immune response and several kinds of metabolic process, which has potential for further exploration of the effect of age on immunotherapy.

### Comparison of Immune Cell Infiltration in Tumor Microenvironment and Potential Relevance of Age in Immunotherapy

**FIGURE 8:**
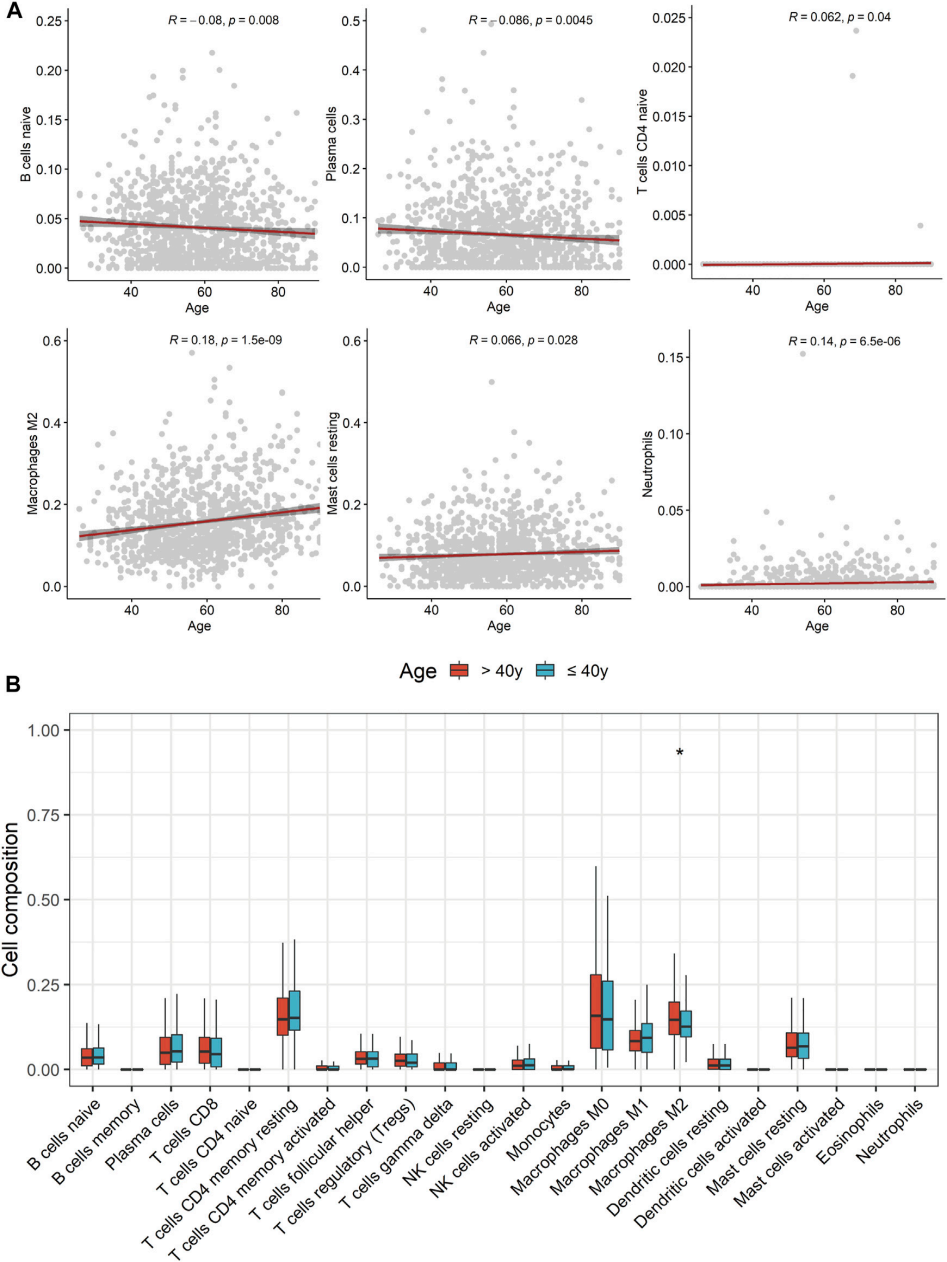
Comparison of tumor immune infiltrating cell types in tumor microenvironment between young and non-young breast cancer samples. **(A)** Correlation scatter diagrams displaying correlation between age and several tumor immune infiltrating cell types. **(B)** Boxplots of the proportions of 22 immune infiltrating cells between two cohorts.

Based on the result of CIBERSORT algorithm, the relevance of age and tumor immune infiltrating cell types were elucidated and the distribution of tumor immune infiltrating cells in TME were compared between young and non-young breast cancer groups in TCGA-BRCA. Correlation analyses revealed that age was negatively correlated with naive B cells and plasma cells while positively correlated to naive CD4+ T cells, M2 macrophages, resting mast cells and neutrophils (Figure 8A). Moreover, we further determined that M2 macrophages were significantly enriched in non-young breast cancer compared to young breast cancer in CIBERSORT analysis result (Figure 8B). Besides, xCell analysis result unveiled that class-switched memory B cells, M2 macrophages, osteoblasts and preadipocytes were abundantly distributed in the TME of non-young breast cancer while common lymphoid progenitor (CLP) cells, keratinocytes and Th2 cells were predominantly enriched in the TME of young breast cancer (Supplementary Figure S2B). The outcomes unveiled that age may remarkably suppress or strengthen the distribution of specific immune cell types, thus potentially affecting the response to immunotherapy.

## Discussion

Currently, breast cancer is the most frequently diagnosed malignancy worldwide (Sung et al., 2021). Metastasis remains the leading cause of breast cancer-related deaths, and the 5-year survival of patients with metastatic breast cancer is only approximately 25% (Valastyan and Weinberg, 2011). Among all breast cancers, young breast cancer is prone to having an aggressive molecular subtype, which predisposes them to elevated breast cancer-related mortality compared to their older counterparts (Gnerlich et al., 2009; Partridge et al., 2016). Therefore, elucidating and comparing the metastatic patterns and survival of breast cancer between young and non-young women is important. The current study, to the best of our knowledge, reports for the first time that young and non-young breast cancer patients present with different metastatic patterns. We also found that young age was a negative prognostic factor, particularly for HR+/HER2- breast cancer. The findings of this study may guide personalized cancer treatment and provide a framework for future clinical trials. The findings of this study may guide personalized cancer treatment and provide a framework for future clinical trials.

In our study, the metastatic rates of bone, liver and DLN in the young cohort were significantly higher than those in the non-young cohort. The impact of molecular subtypes on the metastatic sites in young and non-young patients in our study also showed that the percentages of HER2+ and triple-negative subtypes were much higher in the young cohort than in the non-young cohort. These results are consistent with the findings that young breast cancer is associated with an aggressive phenotype (Azim et al., 2012; Narod, 2012; Azim and Partridge, 2014; Rosenberg et al., 2015). In our study, bone was the most frequent site of metastasis of breast cancer, especially for HR+/HER2– tumors. The percentages of HER2+ subtypes were high among patients with liver and brain metastasis, and the percentage of TNBC was significantly increased in patients with visceral metastasis compared with those with bone metastasis in both cohorts. The above results were consistent with previous studies demonstrating that the metastatic behavior of different breast cancer subtypes differs from each other (Foulkes et al., 2010; Kennecke et al., 2010).

The results from our study showed that more than 1 in 4 patients with metastasis developed multisite metastasis. Consistent with reports in previous studies, two-site metastasis was the most common pattern, followed by three-site, four-site, and five-site metastasis (Wang et al., 2020). The most frequent two-site metastatic combination was bone and liver for the young cohort, whereas it was bone and lung for the non-young cohort. For three-site metastasis, the most common combination was bone, liver and DLN for the young cohort, while the most common combination was bone, liver and lung for the non-young cohort. Young patients with bone metastasis had a higher co-metastasis rate of the liver than others. However, for non-young patients with bone metastasis, the co-metastasis rate of the lung is significantly higher than that of other patients. The co-metastasis rate of bone is much higher than that of other sites for patients with brain, liver, lung and DLN metastasis. The differences in multisite metastasis and co-metastasis patterns between young and non-young cohorts should be taken into account in the clinical management of metastatic breast cancer.

Young breast cancer has long been considered to be associated with less favorable outcomes than older breast cancer (Anders et al., 2009). In a study by Anders et al., young breast cancer (≤45 years) showed a trend toward inferior disease-free survival (Anders et al., 2008). However, the effect of age on survival may vary by tumor subtype. In the HERA trial, the benefits and outcomes from anti-HER2 treatment appear similar for women aged ≤ 40 versus those aged > 40 years (Partridge et al., 2013). In Partridge’s study of 17,575 patients with stage I to III breast cancer, young age (≤40 years) was found to be particularly associated with significant increases in the risk of breast cancer-related death among women with luminal A (hazard ratio, 2.1) and luminal B (hazard ratio, 1.4) tumors (Partridge et al., 2016). The results of the current study demonstrated that young age is a negative prognostic factor, particularly for HR+/HER2- breast cancer (hazard ratio, 1.55), which is consistent with the results of previous studies (Partridge et al., 2016; Fu et al., 2019). Our data support the notion that the effect of young age on the survival of breast cancer varies by molecular subtype.

As for the result of dissecting distribution of tumor immune infiltrating cell types and correlation of age and these cells, age was negatively correlated with naive B cells and plasma cells while positively relevant to naive CD4+ T cells, M2 macrophages, resting mast cells and neutrophils. In addition, M2 macrophages were significantly enriched in non-young breast cancer than young breast cancer. Different from classic M1 macrophages with pro-inflammatory response, M2 macrophages shows an anti-inflammatory phenotype and are found in parasitic infection, allergy, tissue remodelling, waste elimination processes following acute phase inflammation, and tumor development (Mahbub et al., 2011). There have been a host of studies implying that aging macrophages become increasingly skewed towards immunosuppression. For example, peritoneal and splenic macrophages from elderly mice are less responsive to pro-inflammatory stimuli (LPS and IFN-γ) compared to those from young mice (Mahbub et al., 2012). Besides, elderly macrophages stimulated with anti-inflammatory IL-4 and IL-13 stimuli increase production of TGF-β (Jackaman et al., 2014). Furthermore, M2-like macrophages have been proved to promote abnormal angiogenesis in age-related diseases including cancers (Kelly et al., 2007). In detail, elderly M2 macrophages demonstrated a pro-angiogenic phenotype with greater upregulation of IL-10, alongside reduced FasL, IL-12 and TNF-α expression following retina injury as a result that elderly mice were more susceptible to injury-associated angiogenesis. In a nutshell, in non-young breast cancer patients, it is more likely for them to gain an immunosuppressive TME that may obtain inferior response for immunotherapy than young breast cancer.

This study nevertheless has several limitations that should be noted. First, the key disadvantage of this analysis is its retrospective nature, and we cannot completely rule out the impact of selection bias. Second, there was only information on five main sites (bone, brain, liver, lung and DLN) for metastasis in the SEER database, although these five sites accounted for 95.7% of all patients with metastasis. Data on patients with rare metastasis or metachronous metastasis were missing from SEER. Third, women of white and black race constituted most of our study population. Thus, caution should be taken when applying our results to Asian and other ethnic cohorts. Furthermore, we were unable to collect sufficient information that appears to be significant prognostic factors for breast cancer, such as BRCA1/2 mutations. The response to immunotherapy in young and non-young breast cancer remains validation in other cohort of breast cancer patients with immunotherapy. Future well-designed studies are warranted to further validate our findings.

In conclusion, we have demonstrated in a large-scale retrospective analysis that young and non-young breast cancer patients present with different metastatic patterns. The effect of young age on the survival of breast cancer varies by molecular subtype. Young age is a negative prognostic factor, particularly for HR+/HER2- breast cancer. The young breast cancer patients may gain better response to immunotherapy due to immune activated TME than non-young breast cancer. The results of this study may guide individualized treatment for breast cancer by age and provide information for future studies to investigate the benefits of age-informed management.

## Data Availability

The datasets presented in this study can be found in online repositories. The names of the repository/repositories and accession number(s) can be found in the article/Supplementary Material.
