# Supplementary figures and images for: Comparisons of Metastatic Patterns, Survival Outcomes and Tumor Immune Microenvironment Between Young and Non-Young Breast Cancer Patients
Source: Front Cell Dev Biol. 2022 Jul 14;10:923371. doi: 10.3389/fcell.2022.923371 (PMC9329535; doi:10.3389/fcell.2022.923371)

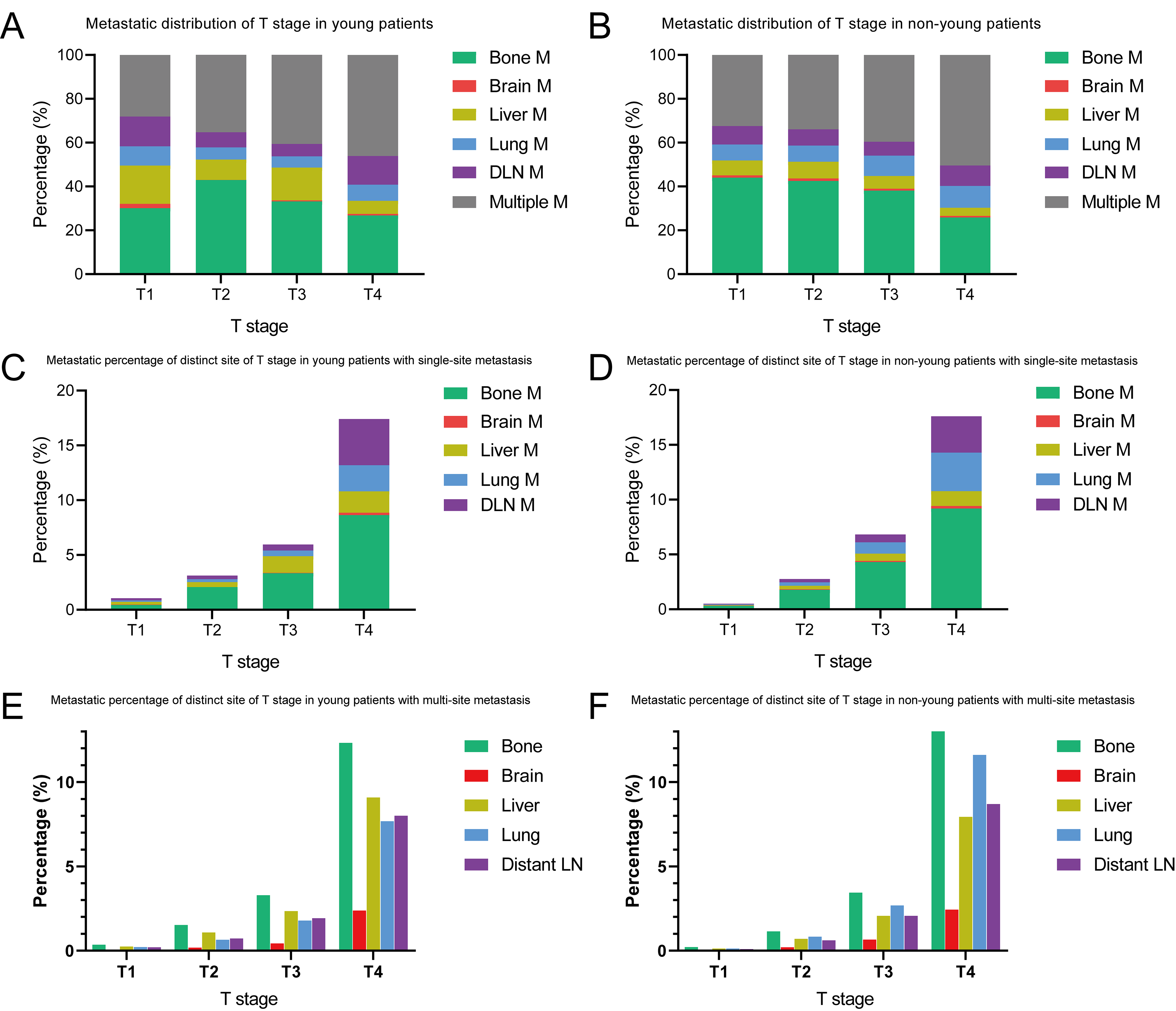

Supplement: Supplementary file 1 [file Image2.TIF]

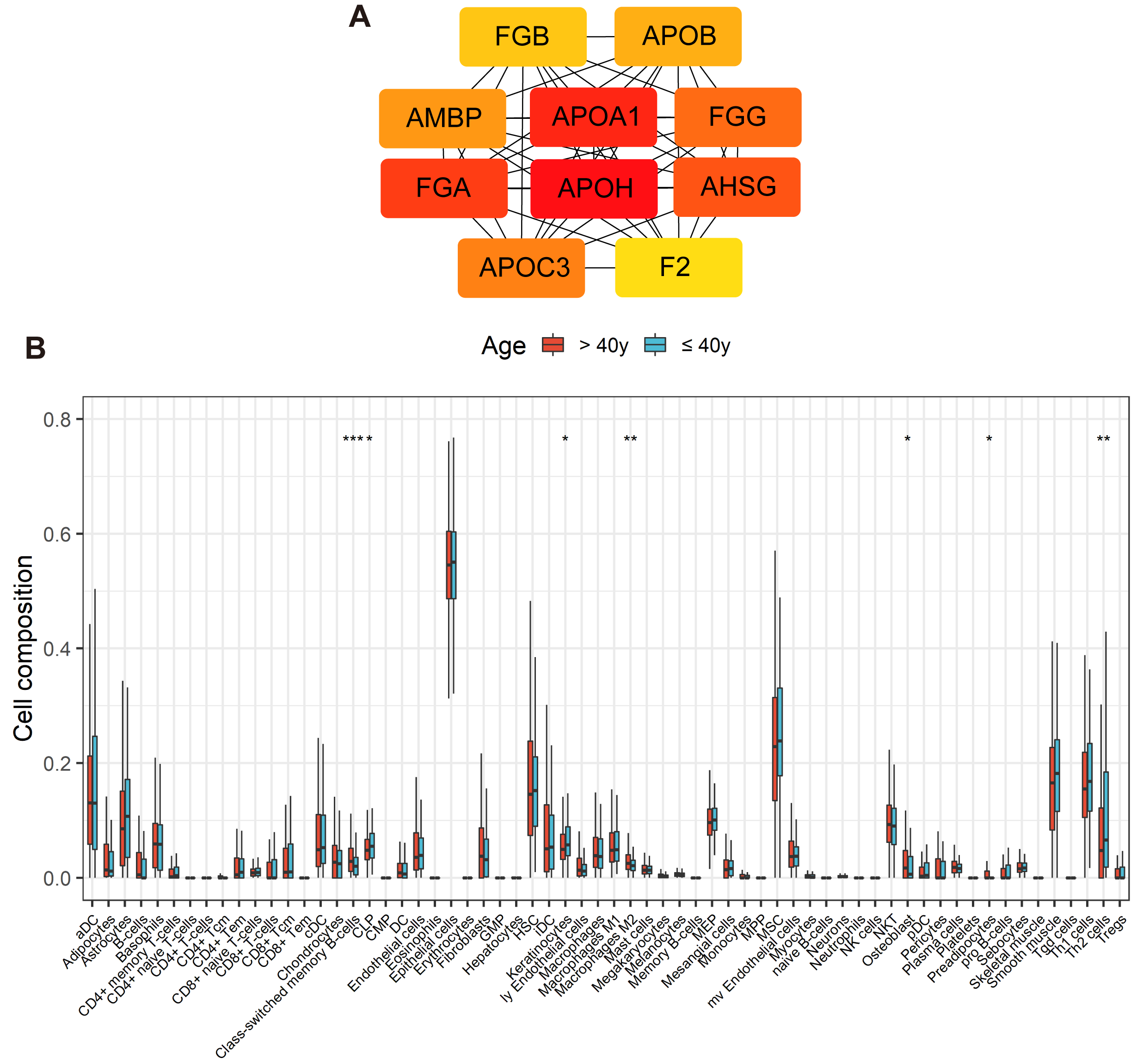

Supplement: Supplementary file 2 [file Image1.TIF]
