# Supplementary material for: Comparisons of Metastatic Patterns, Survival Outcomes and Tumor Immune Microenvironment Between Young and Non-Young Breast Cancer Patients
Source: Front Cell Dev Biol. 2022 Jul 14;10:923371. doi: 10.3389/fcell.2022.923371 (PMC9329535; doi:10.3389/fcell.2022.923371)
Supplement: Supplementary file 3 [file DataSheet1.PDF]

Supplementary Material

Supplementary table 1. Univariate and multivariate analysis of breast-specific survival for patients with hormone receptor-positive/human epidermal growth factor receptor 2-negative tumors.

	Univariate		Multivariate	
	HR (95% CI)	P	HR (95% CI)	P
Young/Non-young		< 0.001		0.001
Non-young	Reference		Reference	
Young	1.55 (1.45-1.64)		1.12 (1.05-1.19)	
Race		< 0.001		
White	Reference			
Black	1.90 (1.82-1.99)			
Asian or Pacific Islander	0.82 (0.77-0.88)			
American Indian/Alaska Native	1.17 (0.95-1.43)			
Unknown	0.29 (0.19-0.45)			
Histology		< 0.001		
Invasive carcinoma	Reference			
Favorable	0.37 (0.32-0.43)			
Metaplastic	4.44 (3.55-5.55)			
Others	1.64 (1.58-1.71)			
Grade		< 0.001		< 0.001
I	Reference		Reference	
II	2.64 (2.49-2.80)		1.67 (1.58-1.78)	
III	7.07 (6.66-7.50)		3.34 (3.14-3.55)	
IV	6.64 (4.98-8.85)		2.73 (2.05-3.65)	
Unknown	9.88 (9.19-10.63)		2.59 (2.40-2.80)	
T stage		< 0.001		< 0.001
1	Reference		Reference	
2	4.80 (4.59-5.02)		2.76 (2.63-2.89)	
3	10.50 (9.95-11.09)		4.18 (3.93-4.44)	
4	30.56 (28.98-32.21)		6.08 (5.70-6.49)	
Others	17.47 (16.29-18.74)		4.37 (3.98-4.79)	
N stage		< 0.001		< 0.001
0	Reference		Reference	
1	3.49 (3.35-3.63)		1.70 (1.63-1.78)	
2	6.47 (6.13-6.83)		2.26 (2.13-2.39)	
3	15.87 (15.13-		3.17 (2.98-	

	16.65)		3.37)	
Unknown	9.59 (8.89-10.35)		2.11 (1.91-2.32)	
Bone metastasis		< 0.001		< 0.001
No	Reference		Reference	
Yes	22.13 (21.31-22.98)		5.65 (5.37-5.94)	
Brain metastasis		< 0.001		< 0.001
No	Reference		Reference	
Yes	34.62 (30.88-38.81)		1.91 (1.69-2.15)	
Liver metastasis		< 0.001		< 0.001
No	Reference		Reference	
Yes	28.35 (26.63-30.18)		2.27 (2.11-2.44)	
Lung metastasis		< 0.001		< 0.001
No	Reference		Reference	
Yes	21.39 (20.24-22.60)		1.45 (1.36-1.55)	
DLN metastasis		< 0.001		
No	Reference			
Yes	17.40 (16.33-18.55)			

Abbreviations: HR: hazard ratio; CI: confidence interval; HR: hormone receptor; HER2: human epidermal growth factor receptor-2; DLN: distant lymph node.

SUPPLEMENTARY FIGURE LEGEND

Supplementary Figure 1: Metastatic patterns of young and non-young cohorts in diverse T stages. (A) Metastatic distribution of T stage in young breast cancer patients. (B) Metastatic distribution of T stage in non-young breast cancer patients. (C) Metastatic percentage of distinct site of T stage in young patients with single-site metastasis. (D) Metastatic percentage of distinct site of T stage in non-young patients with single-site metastasis. (E) Metastatic percentage of distinct site of T stage in young patients with multi-site metastasis. (F) Metastatic percentage of distinct site of T stage in young patients with multi-site metastasis.

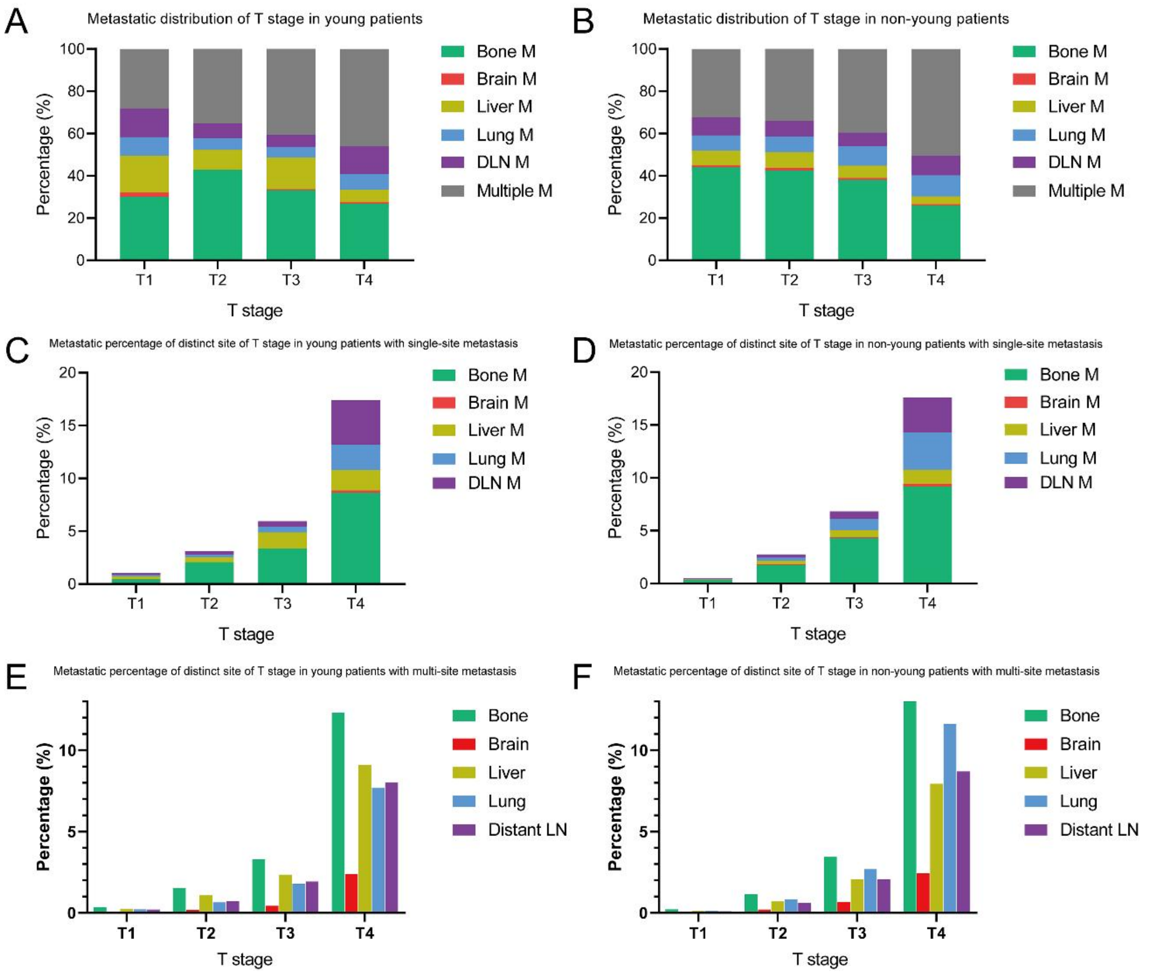

Supplementary Figure 2: (A) PPI network based on DEGs. (B) xCell analysis result in non-young and young cohorts.

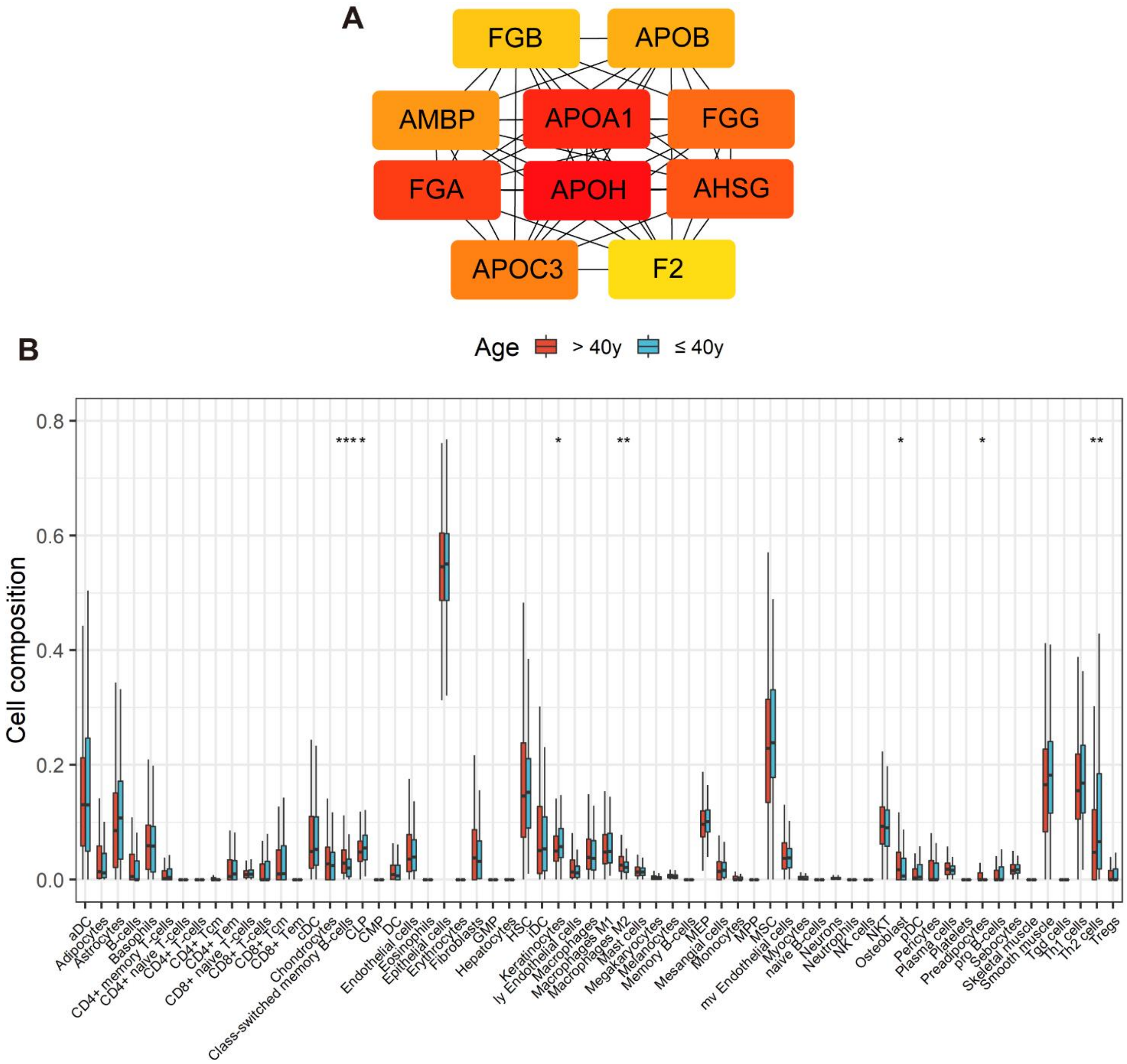
A
FGB
APOB
AMBP
APOA1
FGG
FGA
APOH
AHSG
APOC3
F2
B
Age > 40y ≤ 40y
Cell composition
0.0
0.2
0.4
0.6
0.8
****
*
**
*
*
**
aDC
Adipocytes
Astrocytes
B-cells
Basophils
CD4+ memory T-cells
CD4+ naive T-cells
CD4+ T-cells
CD4+ Tcm
CD4+ Tem
CD8+ naive T-cells
CD8+ T-cells
CD8+ Tcm
CD8+ Tem
cDC
Chondrocytes
Class-switched memory B-cells
CLP
CMP
DC
Endothelial cells
Eosinophils
Epithelial cells
Erythrocytes
Fibroblasts
GMP
Hepatocytes
HSC
iDC
Keratinocytes
ly Endothelial cells
Macrophages
Macrophages M1
Macrophages M2
Mast cells
Megakaryocytes
Melanocytes
Memory B-cells
MEP
Mesangial cells
Monocytes
MPP
MSC
mv Endothelial cells
Myocytes
naive B-cells
Neurons
Neutrophils
NK cells
NKT
Osteoblast
pDC
Pericytes
Plasma cells
Platelets
Preadipocytes
pro B-cells
Sebocytes
Skeletal muscle
Smooth muscle
Tgd cells
Th1 cells
Th2 cells
Tregs
